# Modified 3D-Controlled Inside-Out Compression Screw Fixation Technique in Posterior Malleolar Fractures: A Narrative Review and Case Report

**DOI:** 10.3390/jcm15010154

**Published:** 2025-12-25

**Authors:** Johannes Wunder, Leander Gaul, Johannes Gabel, Ahmet Mestan, Christian von Rüden

**Affiliations:** 1 Department of Trauma Surgery, Orthopaedics and Hand Surgery, Klinikum Weiden, University of Regensburg Teaching Hospital, Söllner Str. 16, 92637 Weiden, Germany; 2 Department of Trauma Surgery, BG Unfallklinik Murnau, Professor-Küntscher-Str. 8, 82418 Murnau, Germany

**Keywords:** ankle fracture, syndesmosis, tibia, internal fracture fixation, three-dimensional imaging, minimally invasive surgical procedure

## Abstract

Fractures of the posterior malleolus are key determinants of ankle stability and long-term functional outcome in complex ankle injuries. The posterolateral rim fragment represents a bony avulsion of the posterior syndesmotic complex. Anatomical reduction of this fragment restores the fibular incisura, posterior tibiotalar stability, and syndesmotic integrity. Based on a geriatric case of a trimalleolar ankle fracture with a Bartoníček type 2 posterior malleolar component, this review describes a modified minimally invasive inside-out fixation technique performed under intraoperative three-dimensional imaging. The posterior malleolar fragment was stabilized using a posterior-to-anterior headless double-threaded compression screw. The medial malleolus was fixed with two parallel partially threaded cannulated cancellous screws, and the distal fibular fracture was stabilized using a reamed intramedullary locking nail. The surgical technique, potential complications, and postoperative management are described in detail. This approach combines the biomechanical advantages of direct posterior malleolar fixation with minimal soft-tissue disruption, providing a stable and reliable construct for the treatment of complex ankle fractures, particularly in geriatric patients and in those with compromised soft-tissue conditions.

## 1. Introduction

Fractures involving the posterior malleolus play a pivotal role in determining ankle stability and long-term functional outcome. The posterolateral rim fragment represents a bony avulsion of the posterior syndesmotic complex. It is well established that involvement of the posterior malleolus—regardless of fragment size—is associated with an increased risk of post-traumatic ankle osteoarthritis [1,2]. Anatomical reduction of the posterolateral rim fragment restores the fibular incisura, the posterior distal tibial articular surface, posterior tibiotalar stability, and the integrity of the posterior syndesmotic complex [3]. Accurate reconstruction of the fibular notch is essential for anatomical reduction of the distal fibula, thereby preventing malreduction and reducing the need for secondary syndesmotic fixation [4,5]. Historically, surgical fixation was recommended when the posterior malleolar fragment involved more than 25% of the distal tibial articular surface on lateral radiographs [6]. However, this criterion frequently underestimated fragment size and complexity. With the widespread use of computed tomography (CT), fracture morphology was reassessed in up to 44% of cases, resulting in changes to the surgical strategy [7]. Consequently, CT imaging has become indispensable for preoperative evaluation of posterior malleolar fractures. Recent advances in intraoperative imaging have enabled direct assessment of reduction quality using three-dimensional (3D) fluoroscopy, thereby reducing the need for postoperative CT scans and potentially avoiding secondary revision procedures. Despite these developments, the optimal surgical treatment of posterior malleolar fractures remains controversial. Patient-reported functional outcomes correlate closely with fracture severity and morphology [8]. Direct fixation of the posterior malleolar fragment has been demonstrated to provide superior biomechanical stability compared with indirect fixation techniques or isolated syndesmotic transfixation [9,10,11]. Fracture morphology and associated injuries therefore guide the choice of surgical approach [10]. Posterior malleolar fixation may be achieved via open reduction and internal fixation using screws and/or plates or via minimally invasive percutaneous reduction and screw fixation. Traditionally, percutaneous fixation was performed using an indirect anterior-to-posterior traction screw. However, concerns regarding inadequate reduction control and suboptimal interfragmentary compression were raised. Subsequent modifications introduced posterior-to-anterior compression screws to overcome these biomechanical limitations. Building on these concepts, several authors have described refinements of the technique, including the posterior-to-anterior inside-out approach [12].

This review presents a further modification of the inside-out technique, incorporating direct percutaneous posterior-to-anterior screw fixation of the posterior malleolar fragment using intraoperative 3D fluoroscopic control and headless double-threaded compression screws.

## 2. Preoperative Diagnostics

Standard diagnostic evaluation of ankle fractures includes conventional biplanar radiographs comprising mortise and lateral views. In cases of suspected Maisonneuve injury, additional imaging of the knee and lower leg in two planes is mandatory [5,9]. Nevertheless, precise visualization of subtle articular incongruities, step-offs, and implant malpositioning remains challenging due to the complex convex and concave geometry of the ankle joint surfaces. Inadequate intraoperative visualization often necessitates postoperative CT imaging, particularly as unrecognized malreduction and implant malpositioning account for a substantial proportion of revision surgeries and are associated with inferior clinical outcomes [13,14]. With increasing availability of intraoperative 3D imaging, reduction quality and implant placement can now be assessed immediately during surgery. This development significantly reduces the need for postoperative CT scans and helps prevent avoidable revision procedures.

## 3. Intraoperative 3D Fluoroscopic Control

The introduction of intraoperative 3D fluoroscopy has substantially improved the accuracy of fracture reduction and implant positioning in ankle surgery [15,16]. Conventional two-dimensional fluoroscopy frequently fails to adequately depict complex articular and syndesmotic relationships, particularly in the posterior and medial regions of the ankle [6,17]. In contrast, 3D C-arm fluoroscopy enables multiplanar assessment of reduction quality and hardware positioning directly in the operating room, thereby reducing the risk of undetected malreduction or intra-articular screw penetration [18,19]. Accurate assessment of the posterior key fragment, intercalary fragments, fibular incisura, and associated fracture components is essential [20,21]. Several clinical studies have demonstrated that intraoperative 3D imaging leads to relevant intraoperative corrections in up to one-third of cases, even when conventional fluoroscopy suggests adequate reduction [20]. By providing immediate feedback, this technology allows for correction of incongruent reductions or misplaced implants prior to wound closure, potentially lowering revision rates and improving functional outcomes. However, intraoperative 3D imaging has limitations. Image resolution and field of view remain inferior to postoperative CT, and the absence of a contralateral reference can hinder detection of subtle rotational malalignment [22]. Additionally, image acquisition and reconstruction increase operative time and radiation exposure, although modern low-dose protocols have reduced these concerns [19]. Cost and limited availability further restrict widespread implementation. Despite these limitations, intraoperative 3D imaging represents a major advancement in the management of complex ankle fractures—particularly those involving the posterior malleolus and syndesmosis—by promoting accuracy-driven fracture surgery [2,5,7,23]. In the present setting, intraoperative 3D fluoroscopy was specifically used to assess (1) residual articular step-off ≥ 1 mm, (2) posterior malleolar displacement > 1–2 mm, and (3) intra-articular screw penetration, all of which were predefined as thresholds necessitating immediate correction.

## 4. Classification

The increasing complexity of ankle fractures has brought cross-sectional imaging to the forefront of preoperative assessment [24]. Conventional radiograph-based classification systems often lack reliability. Multislice CT imaging with 3D reconstructions enables precise characterization of fracture morphology. In this context, the authors employ the CT-based classification system described by Bartoníček et al., which distinguishes five fracture types and provides guidance for surgical decision-making [9].

## 5. Case Description

**Figure 1:**
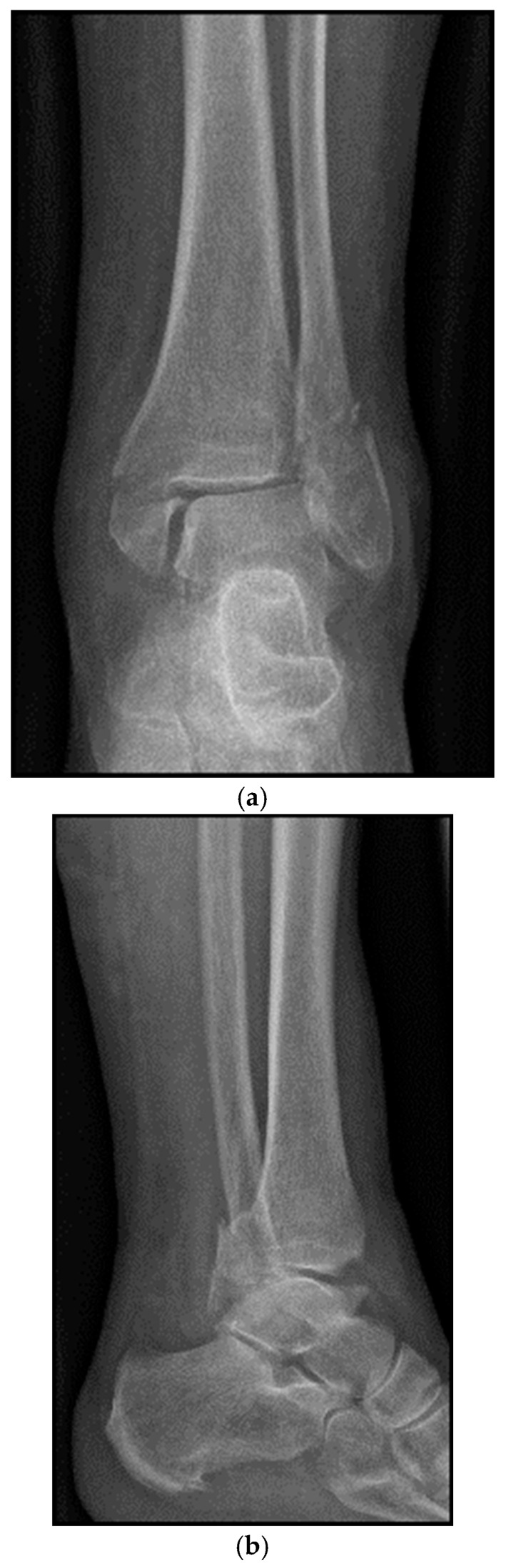

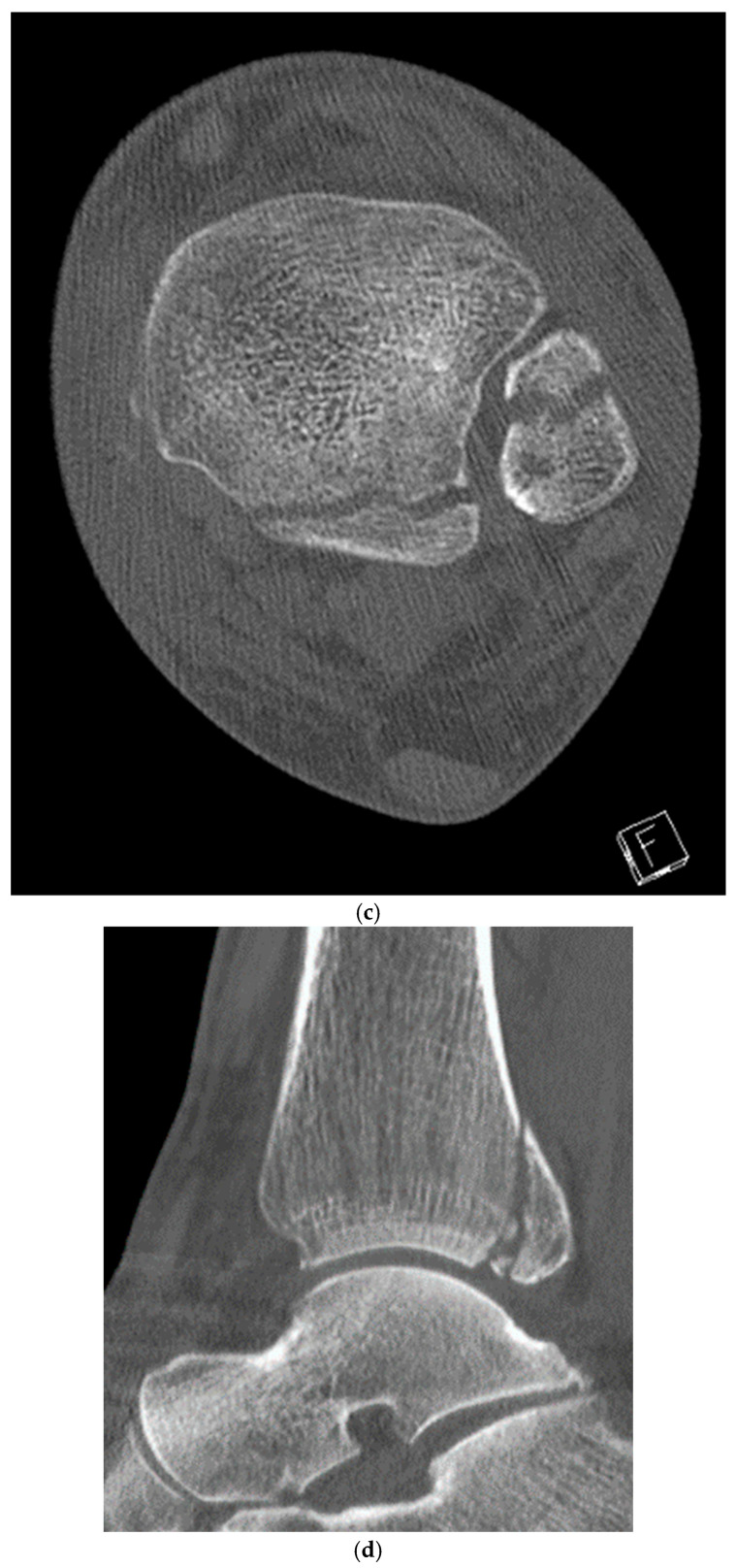
(**a**–**d**): Trimalleolar ankle fracture: (**a**) anteroposterior and (**b**) lateral radiographs; (**c**) axial and (**d**) sagittal CT views demonstrating type 2 posterior malleolar fracture with small intercalary fragments.

An 83-year-old female patient sustained a trimalleolar ankle fracture with a Bartoníček type 2 posterior malleolar fracture involving a posterolateral fragment and the fibular incisura after slipping on a smooth surface (Figure 1a–d). Relevant comorbidities included reduced bone quality, compromised soft-tissue conditions with parchment-like skin, and adult-onset diabetes mellitus (HbA1c 7.7%).

### 5.1. Surgical Considerations

Complex unstable and displaced ankle fractures pose significant challenges due to fracture morphology, articular involvement, and vulnerable soft-tissue coverage [25,26]. Increasing patient age and associated comorbidities—including peripheral arterial disease, diabetes mellitus, osteoporosis, and chronic steroid use—further complicate surgical management. Restoration of physiological axial, torsional, and longitudinal alignment is essential for functional recovery and prevention of post-traumatic osteoarthritis but may be difficult in the presence of compromised soft tissues.

With advancing age, pronation–adduction type fractures according to the Lauge–Hansen classification become more prevalent. These injuries are frequently underestimated yet highly unstable and often associated with irregular fracture patterns at the syndesmotic level [27]. In such scenarios, minimally invasive fixation techniques that limit surgical trauma while permitting anatomical reduction are particularly advantageous.

### 5.2. Inside-Out Fixation of the Posterior Malleolus Using a Headless Double-Threaded Compression Screw

**Figure 2:**
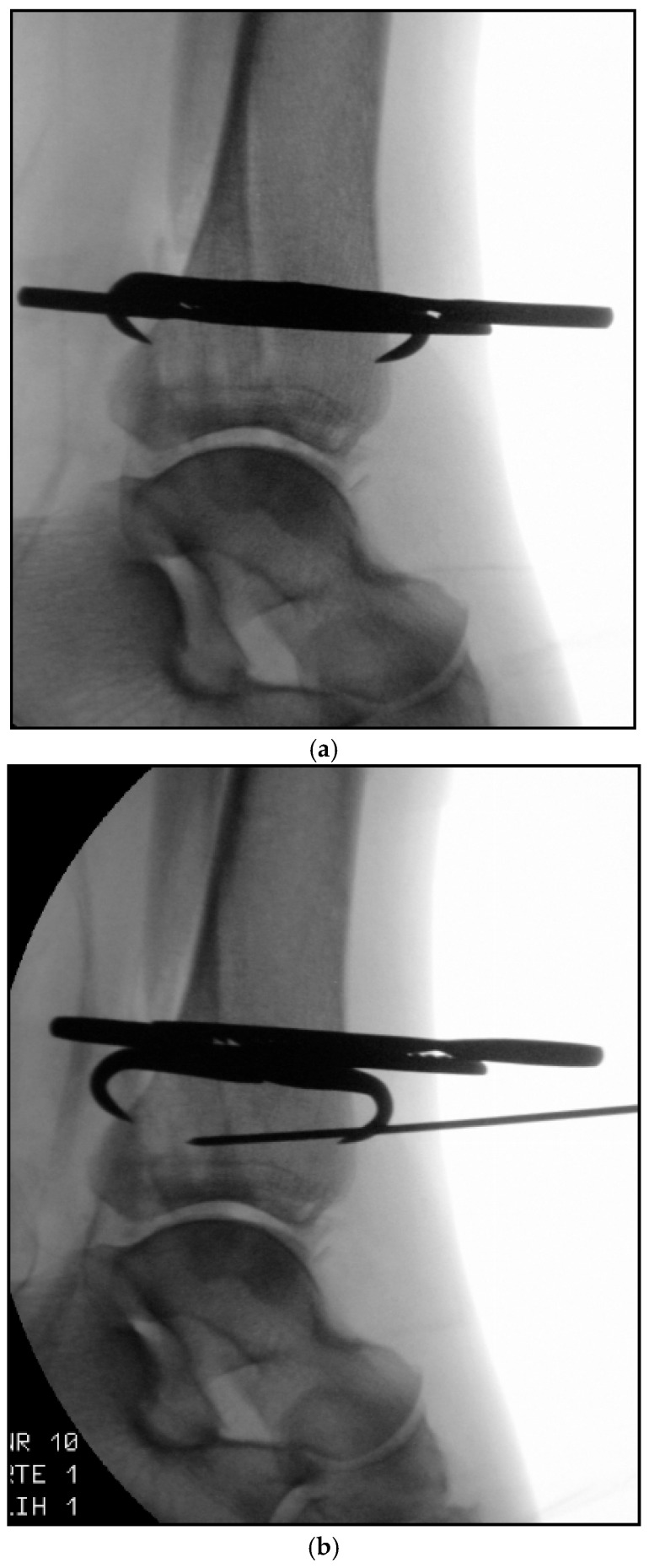

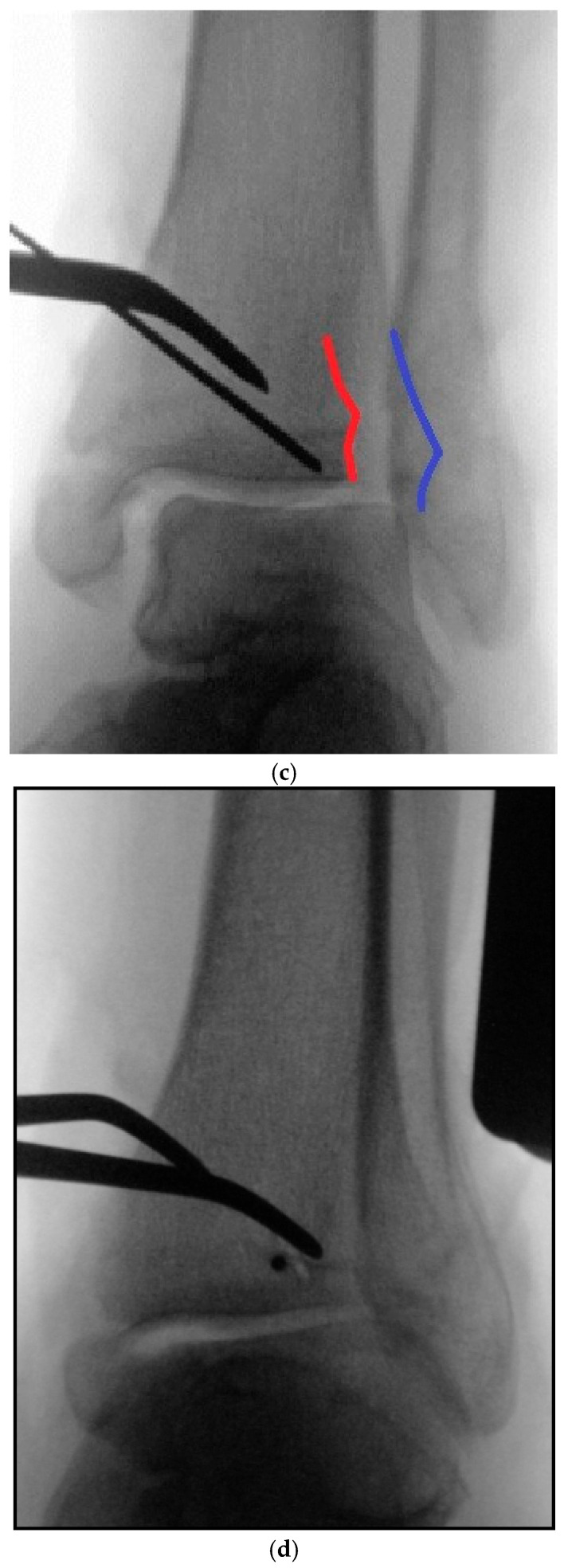

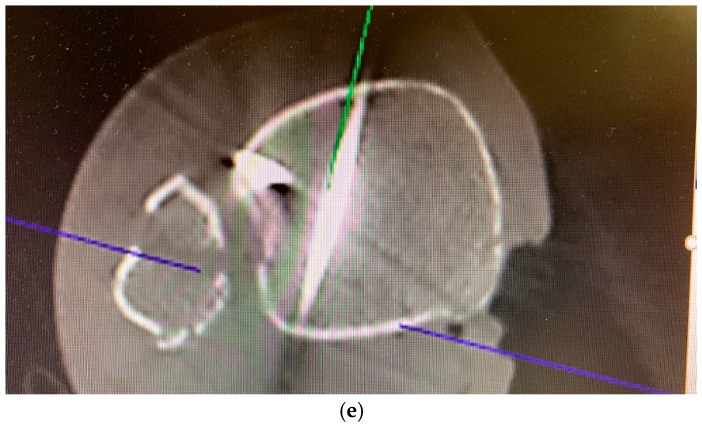
(**a**–**e**): (**a**) Temporary retention with pointed reduction clamp; (**b**) insertion of the 1.6 mm guidewire from anterior to posterior; (**c**) mortise view with marked boundaries of the fibular notch (red line: posterior border, blue line: anterior border); (**d**) punctate end view confirming extraincisural trajectory; (**e**) intraoperative 3D fluoroscopy demonstrating the correct course of guide wire.

Surgery was performed under spinal anesthesia with the patient in the supine position following single-shot antibiotic prophylaxis. After sterile preparation and draping, fixation of the posterior malleolus was undertaken as the initial step. A modified posteromedial approach was used. The skin incision followed the course of the tibialis posterior tendon along the posterior tibial border, curving anteriorly toward the medial malleolar tip. After sharp dissection of the subcutaneous tissue, the flexor retinaculum was exposed and incised longitudinally over the tibialis posterior tendon. The tendon was mobilized bluntly and, together with the flexor digitorum longus tendon, neurovascular bundle, and flexor hallucis longus tendon, retracted dorsolaterally to expose the posterior tibial surface. Following careful subperiosteal exposure, the posterior malleolar fracture was visualized, interposed fragments were removed, and the fragment was anatomically reduced and temporarily stabilized using a pointed reduction clamp (Figure 2a). After confirming anatomical reduction, the guidewire entry point was defined proximal to the physeal scar in the lateral third of the tibial plafond. This location enabled a posterior-to-anterior screw trajectory perpendicular to the fracture plane, maximizing compression while avoiding intra-articular penetration. Through a 1 cm longitudinal skin incision, the anterior tibial cortex was exposed. A 1.6 mm guidewire for a 4.5 mm headless double-threaded compression screw (Cannulated Compression Headless Screw; DePuy Synthes, Oberdorf, Switzerland) was inserted from anteromedial to posterolateral with a proximal-to-distal inclination of approximately 5–10°, under fluoroscopic guidance and protection of the extensor tendons and neurovascular structures using two small Longbeck hooks (Figure 2b–d). After confirming correct guidewire placement using intraoperative 3D fluoroscopy (Figure 2e), screw length was measured.

**Figure 3:**
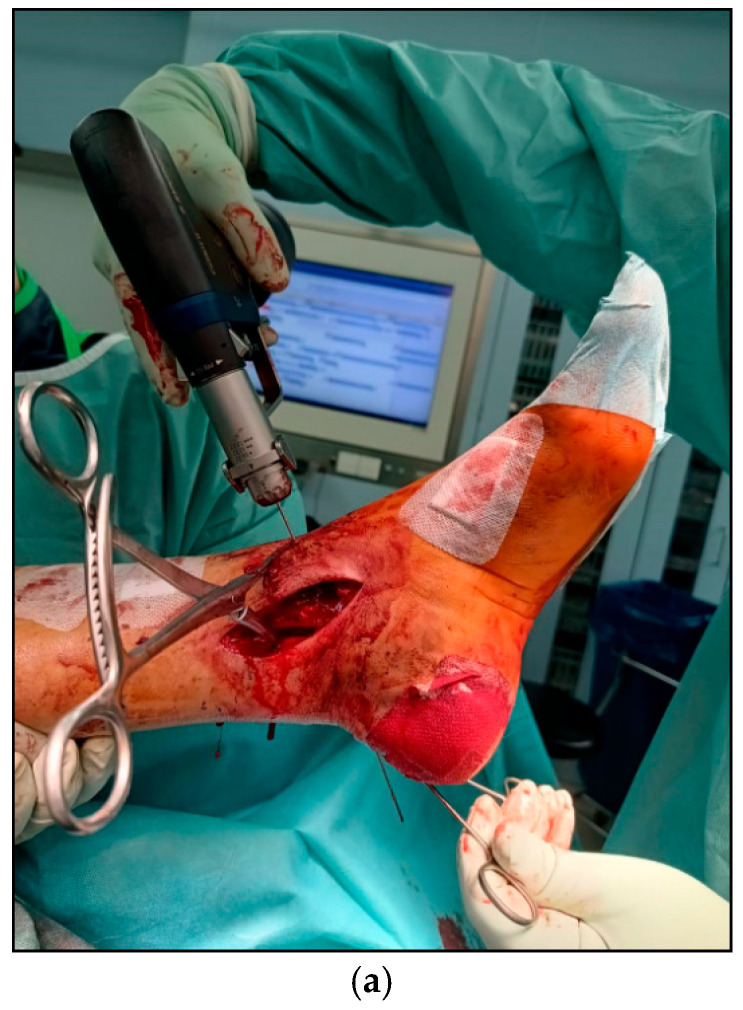

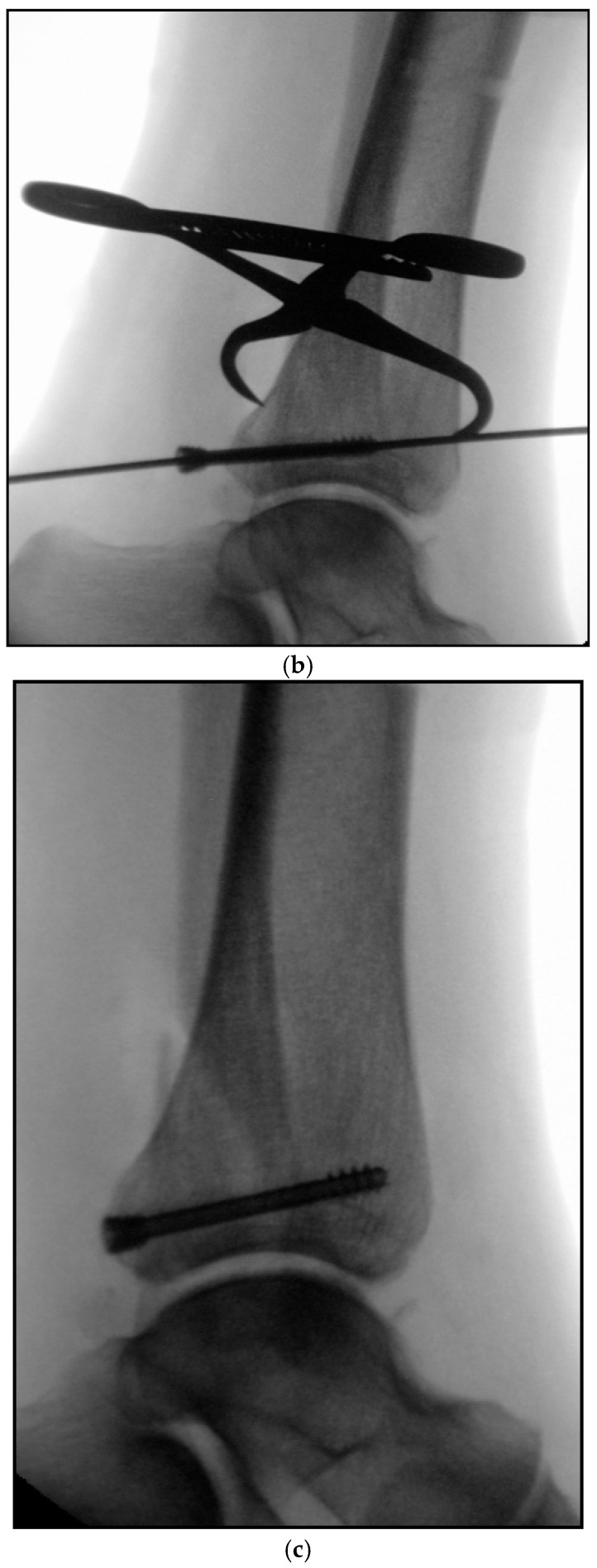
(**a**–**c**): (**a**) Intraoperative clinical view demonstrating reduction of the posterior malleolar fracture using a pointed reduction clamp via modified posteromedial approach. The guidewire has already been advanced dorsally through a subcutaneous corridor created with a clamp; (**b**) lateral fluoroscopic image demonstrating correct insertion of the headless double-threaded screw from posterior to anterior; (**c**) lateral projection following anatomical fixation of the posterior malleolar fragment.

The guidewire was advanced until palpable beneath the skin lateral to the Achilles tendon while the tendon was gently medialized. A small skin incision was made over the wire tip, the wire was withdrawn, and a subcutaneous corridor lateral to the Achilles tendon was created using a clamp to protect the sural nerve. The guidewire was then re-advanced along this tunnel, and a 38 mm, 4.5 mm headless double-threaded compression screw was inserted until one thread engaged the anterior cortex and the other the posterior fragment, achieving stable interfragmentary compression (Figure 3a–c).

### 5.3. Specific Considerations on Double-Threaded Screws in the Management of Complex Ankle Fractures

As demonstrated by biomechanical and anatomical studies, the paraphyseal region exhibits increased cancellous bone density, allowing screws terminating in this area to achieve superior compression and fixation stability [28,29,30]. In this regard, the double-threaded, headless screw has become an increasingly popular fixation device for the treatment of complex ankle fractures, particularly those involving the posterior malleolus. Its characteristic dual-pitch thread design provides controlled interfragmentary compression while avoiding screw head prominence—an advantage in periarticular regions where soft-tissue irritation might hinder recovery [3,31]. This low-profile configuration permits secure fixation through smaller incisions and facilitates earlier postoperative mobilization by minimizing hardware-related discomfort. From a biomechanical view, several studies have demonstrated that direct fixation of the posterior malleolar fragment using headless double-threaded screws enhances rotational stability and restores torsional stiffness more effectively than syndesmotic fixation alone [3,32]. The dual-thread mechanism generates reliable purchase across the fracture line and supports anatomic restoration of the articular surface. Moreover, the headless design reduces the risk of tendon irritation, particularly to the flexor hallucis longus, and helps prevent postoperative adhesions [33,34]. These properties make double-threaded screws especially attractive in the minimally invasive inside-out fixation technique described here, where soft-tissue preservation and limited exposure are key priorities [35]. However, the optimal screw length and thread engagement must be planned carefully to ensure that one thread segment reliably purchases the anterior tibial cortex while the second secures the posterior fragment, thereby generating balanced compression without fragment displacement. Nevertheless, the technique is not completely without drawbacks. Accurate preoperative planning and meticulous intraoperative imaging are essential to prevent malreduction or screw misplacement, which could compromise the incisura or the articular surface [36]. Moreover, hardware removal can be technically challenging, especially when bony integration has occurred, due to the absence of a protruding screw head. Additionally, the relatively high compression forces produced by the dual-thread design may lead to over-compression or fragment displacement in osteoporotic bone, as suggested by recent biomechanical analyses [37]. While current retrospective series have reported favorable radiographic and short-term functional outcomes following the use of headless or double-threaded screw fixation [33], robust prospective trials are still lacking. Future research may aim to clarify whether the theoretical biomechanical advantages of these implants translate into improved long-term outcomes in terms of ankle stability, function, and prevention of post-traumatic osteoarthritis [32].

### 5.4. Medial Malleolar Cancellous Screw Fixation Using the Modified Posteromedial Approach

Following fixation of the posterior malleolus, attention was directed to the medial malleolar fracture through the same posteromedial incision. After subtile periosteal elevation and removal of interposed fragments, the main fragments were reduced and held with a ball spike. Two parallel guidewires were inserted for 4.0 mm partially threaded cancellous screws under biplanar fluoroscopic control, partially overdrilled and definitive screws including washers were placed so that the threads extended up to directly proximal to the physeal scar. This ensured that the threaded segment crossed the fracture line entirely while maximizing compression without causing articular penetration.

### 5.5. Reamed Intramedullary Locking Nail Fixation of the Distal Fibular Fracture

The distal fibular fracture was then stabilized by reamed intramedullary locked nailing (VITUS Fi Fibula Nail System, Marquardt axomed GmbH, Spaichingen, Germany). The intramedullary nail was chosen in order to achieve minimal soft tissue compromise and minimal wound areas on the one hand, and maximum stability through the intramedullary load-bearing element on the other. At the same time, the nail offers maximum rotational stability due to its design and its locking option between the distal fibula and tibia. These properties allow for immediate symptom-oriented mobilization, especially in geriatric patients and patients who are unable to maintain temporary partial weight-bearing for other reasons [38,39]. In addition, intramedullary fixation may better preserve periosteal blood supply due to reduced soft-tissue dissection and has been associated with significantly lower rates of wound-healing complications compared with plate osteosynthesis.

**Figure 4:**
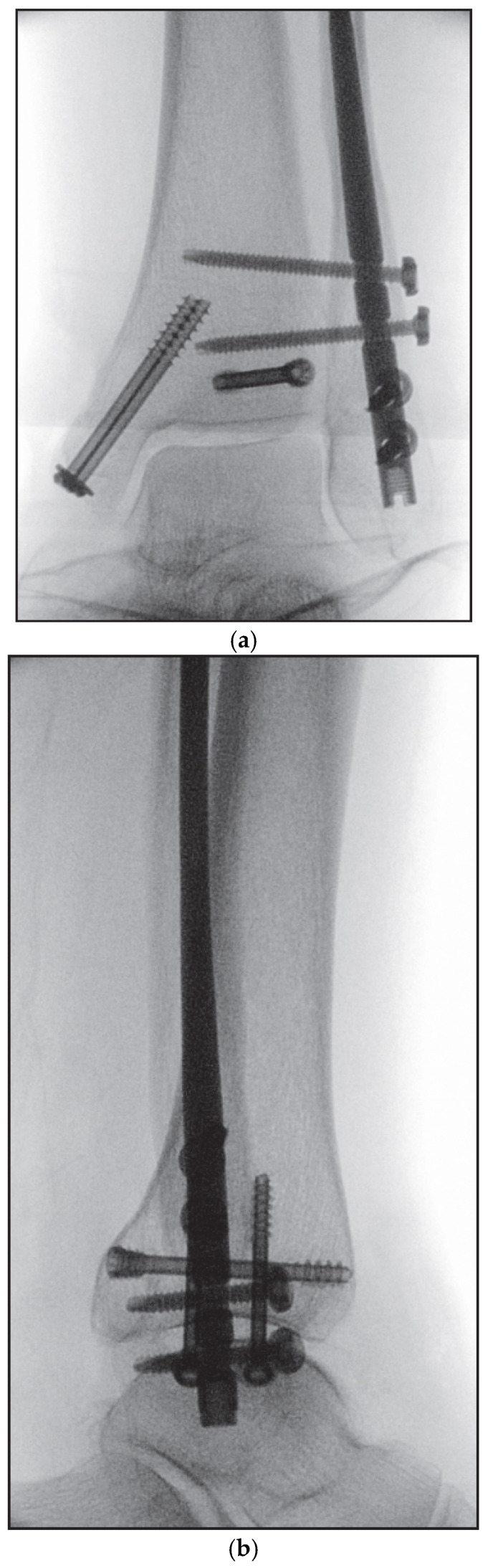

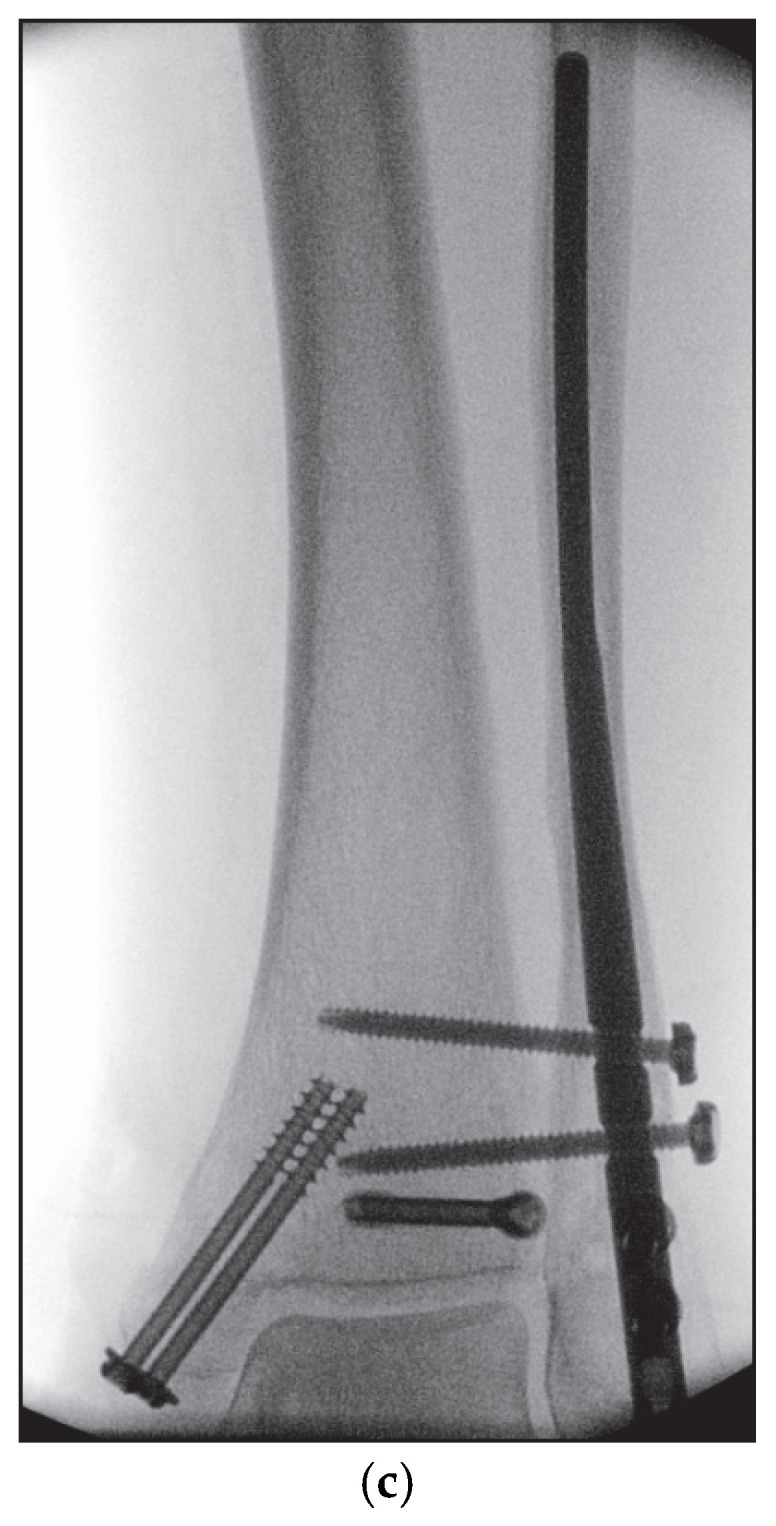
(**a**–**c**): Final intraoperative fluoroscopic images: (**a**) anteroposterior view, (**b**) lateral view, (**c**) mortise view.

The surgical technique is beginning with a short incision from the fibular tip to distally. The subcutaneous tissue was sharply dissected and a protective sleeve was centered. A guidewire was advanced through the sleeve into the medullary canal into the proximal fragment. The entry was opened with a cannulated awl, followed by sequential canal reaming with 6.1 mm reamer in the distal and 3.1 and 3.6 mm reamers in the proximal fragment. Sequential reaming was performed under fluoroscopic monitoring to avoid cortical perforation and to ensure a centered nail path. The fibular nail, mounted on its targeting device, was inserted under fluoroscopic guidance to the intended depth, rotated laterally, and locked with two anterior–posterior and two tricortical syndesmotic screws. An end-cap was not used. Final fluoroscopic documentation was performed in multiple planes (Figure 4a–c). Following thorough irrigation and hemostasis, the tibialis posterior tendon was reduced, the retinaculum was repaired, and layered closure was performed using interrupted Donati sutures. A sterile dressing and a dorsal splint were applied.

## 6. Advantages of the Inside-Out Technique

The inside-out technique offers several advantages over conventional fixation methods. Owing to its minimally invasive nature, this approach is associated with reduced soft-tissue trauma and a lower risk of neurovascular or tendon injury [12]. The flexor hallucis longus tendon is not violated, thereby minimizing the risk of postoperative adhesions. Moreover, the procedure can be performed with the patient in the supine position, which simplifies intraoperative logistics and overall perioperative management. The use of a headless double-threaded compression screw provides rotationally stable fixation and reliable interfragmentary compression, contributing to an anatomically accurate and biomechanically robust reconstruction with minimal surgical exposure. In addition, the posterior-to-anterior screw trajectory aligns the implant perpendicular to the fracture plane, thereby optimizing interfragmentary compression and reducing the risk of secondary displacement [3]. This orientation also overcomes technical limitations associated with anterior-to-posterior screw placement, such as fragment toggling or loss of reduction, which have been reported with indirect fixation techniques. By combining direct fracture visualization with a low-morbidity percutaneous screw path, the inside-out technique enables predictable reduction quality while avoiding extensive posterior soft-tissue dissection.

## 7. Potential Risks and Complications

As with any surgical intervention, general operative risks—including infection, hematoma formation, delayed wound healing, and thromboembolic events—must be considered. In addition, the inside-out fixation technique carries several specific potential complications. A primary concern is injury to the superficial or deep peroneal nerve, the anterior tibial artery, or the extensor tendons during anterior guidewire insertion [40]. Adequate exposure and careful preparation of the anterior tibial cortex, together with the consistent use of a drill sleeve, are essential preventive measures [41,42]. Injury to the sural nerve represents another potential complication during screw insertion. This risk can be mitigated by gentle medialization of the Achilles tendon and by creating a subcutaneous passage within the lateral “safe zone” prior to insertion of the headless double-threaded screw [43,44]. Screw malposition, particularly misplacement within the fibular incisura, is another potential issue and may be avoided through meticulous preoperative planning and intraoperative three-dimensional fluoroscopic control. In the present case, intraoperative 3D imaging proved instrumental in preventing malreduction and implant malposition. Nevertheless, residual syndesmotic instability—including rotational instability—may persist despite stable fixation of the posterior syndesmotic complex. Intraoperative external rotation stress testing, such as the Frick test, facilitates early detection and allows for immediate correction if necessary [45]. If instability persists following posterior fixation, supplemental syndesmotic stabilization using tetracortical screws or a suture-button device may be indicated.

## 8. Postoperative Management

The authors’ postoperative treatment protocol includes immobilization in the intraoperatively applied dorsal lower-leg splint for approximately one week to allow for initial wound healing, with non-weight-bearing mobilization using forearm crutches until soft-tissue consolidation. Subsequently, the patient is transitioned to a walker boot for an additional five weeks. Adjunctive measures include limb elevation, edema control, pain-adapted analgesia, and guideline-based thromboprophylaxis. Physiotherapy focusing on early active range-of-motion exercises of the tibiotalar joint is initiated immediately after splint removal. Inversion and eversion movements are avoided during the initial healing phase to protect the posterior malleolar fixation. Full weight-bearing is generally permitted after six weeks, depending on radiographic evidence of fracture consolidation and patient-reported pain levels.

## 9. Follow-Up

**Figure 5:**
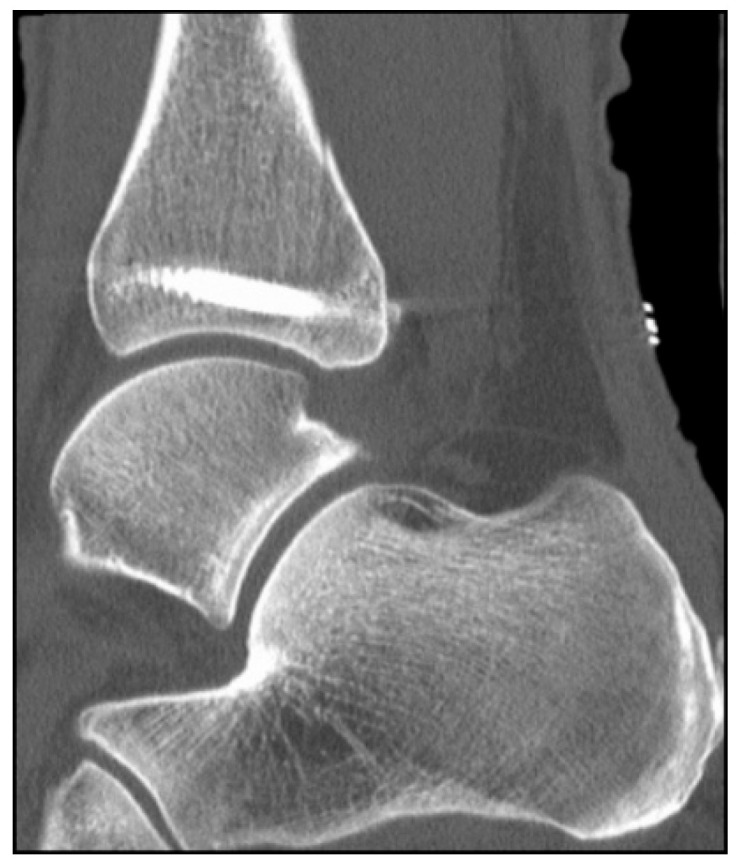
Postoperative CT scan demonstrating optimal screw position and anatomical reduction.

In the present case, partial weight-bearing was not feasible due to the patient’s advanced age and inability to ambulate safely with a walker or to comply with restricted loading. Consequently, early mobilization was performed with pain-adapted full weight-bearing without external immobilization. A postoperative CT scan was obtained prior to hospital discharge (Figure 5).

Functional outcomes and patient-reported measures were assessed using the Foot and Ankle Outcome Score (FAOS; German version, LK 1.0, 2015) and the EQ-5D-5L questionnaire [46]. Ankle range of motion was measured according to the neutral-zero method [47]. At the 12-month follow-up, the patient demonstrated a dorsiflexion/plantarflexion range of motion of 20–0–40°, indicating restoration of functional tibiotalar joint mobility.

Weight-bearing radiographs obtained at 12 months revealed a congruent, step-free tibiotalar articular surface without evidence of progressive post-traumatic osteoarthritis [48]. The osteosynthesis material remained intact. FAOS results demonstrated minimal symptoms across all subscales (Symptoms 0, Stiffness 0, Pain 0, Activities of Daily Living 0, Sports/Recreation 0, Quality of Life 1). The EQ-5D-5L indicated no limitations in mobility, self-care, usual activities, or pain, and no anxiety or depression, with an overall health index score of 95/100.

## 10. Ongoing and Future Research

To address the inherent limitations of a single case report, a retrospective analysis of posterior malleolar fractures treated with the inside-out technique is currently underway. In addition, a prospective comparative study has been initiated to evaluate minimally invasive, 3D fluoroscopy-guided fixation of posterior malleolar fractures versus open reduction and internal plate fixation via the posterolateral approach. These studies aim to provide more robust clinical and radiological evidence regarding the feasibility, accuracy, and functional outcomes of the modified inside-out technique.

## 11. Conclusions

Anatomical reduction and fixation of the posterior malleolus are critical for restoring the fibular incisura and the posterior tibial articular surface, both of which are essential for accurate reduction of the distal fibula. Precise reconstruction of the posterior malleolar fragment also re-establishes posterior tibiotalar stability and preserves the integrity of the posterior syndesmotic complex. Selection of the surgical approach should be guided by fracture morphology and associated soft-tissue or osseous injuries.

Minimally invasive inside-out fixation of the posterior malleolus is particularly suitable when a posteromedial approach is employed and in simple, minimally displaced fracture patterns that allow reliable percutaneous reduction and stabilization. This technique combines the biomechanical advantages of direct posterior fragment fixation with minimal soft-tissue disruption, making it an attractive option for elderly patients and for those with compromised soft-tissue conditions or an elevated risk of wound complications.

Although intraoperative three-dimensional fluoroscopy has inherent technical limitations, it is highly effective in detecting subtle malreductions and implant malpositioning that may be missed on conventional two-dimensional fluoroscopy. In the absence of intraoperative 3D imaging, postoperative computed tomography is essential to identify rotational malalignment, intra-articular screw penetration, intra-articular loose bodies, or marginal articular impaction. When residual incongruity or malalignment is identified, prompt surgical revision is recommended to prevent chronic syndesmotic instability and the subsequent development of post-traumatic ankle osteoarthritis.

## Data Availability

The dataset analyzed during the current work is available from the corresponding author upon reasonable request.
